# Crisis care: tackling the climate and ecological emergency

**DOI:** 10.1192/bjb.2021.33

**Published:** 2021-08

**Authors:** Cate Bailey, Norman A. Poole, Adrian James

**Affiliations:** 1 East London NHS Foundation Trust, UK; 2 Centre for Psychiatry, Queen Mary University and Barts and the London School of Medicine, UK; 3 Royal College of Psychiatrists, UK; 4 Association for Psychoanalytic Psychotherapy in the NHS, UK; 5 St George's Hospital, South West London and St George's Mental Health NHS Trust, UK

**Keywords:** Sustainability, climate emergency, climate crisis, mental health, psychiatrists

## Abstract

The climate crisis is a health crisis; it demands the urgent attention and action of healthcare professionals and organisations. In this issue of the *BJPsych Bulletin*, we consider what the destructive effects of the climate and ecological crisis entail for the mental health of populations, and what the response of psychiatrists, both individual and collective, must be. We also highlight the opportunities and benefits a more sustainable and preventative approach could offer individuals, communities and the planet.

‘We are perilously close to the collapse of civilization, we are all in danger, and only a mobilization of our entire economy and society can protect us’ – Margaret Klein Salamon, activist, psychologist and author.^1^

‘Undoubtedly climate change poses the most profound long-term threat to the health of the nation’ – Simon Stevens, Chief Executive, National Health Service in England.^2^

Without question, human activity throughout the past century and more has led to the destruction of ecosystems on which we depend, and contributed to unprecedented levels of greenhouse gases in our environment. The results of these activities are a disrupted ecology: extreme weather, severe storms, floods, droughts and heatwaves at a frequency and intensity never before seen during the span of human existence. Changing patterns of land use, rising sea levels, destruction of habitats, biodiversity loss, deforestation, soil degradation, plastic waste and air pollution combine with climate change to create food shortages, forced migration, social upheaval and conflict, and increased vulnerability to infectious disease.^3^ The World Health Organization estimates that in the next 20 years, climate change alone will lead globally to 250 000 additional deaths per year.^3^

In this issue of the *BJPsych Bulletin*, we consider what the destructive effects of the climate and ecological crisis entail for the mental health of populations, and what the response of psychiatrists, both individual and collective, must be. We also highlight the opportunities and benefits that a more sustainable and preventative approach could offer individuals, communities and the planet.

## The climate crisis is a health crisis

The climate crisis is a health crisis; it demands the urgent attention and action of healthcare professionals and organisations. We at the *BJPsych Bulletin* share this view with the authors of the *Lancet* Countdown on Health and Climate Change,^4^ editors of the *BMJ*^5,6^ and members of the UK Health Alliance on Climate Change (including the Royal Colleges of Nursing, Physicians, General Practitioners, Emergency Medicine and Surgeons; Faculty of Public Health; Royal Society of Medicine and Royal College of Psychiatrists (RCPsych)).^7^

While the RCPsych first appointed an Associate Registrar for Sustainability in 2015, supported by an active committee, it has recently prioritised its focus in this area. In a new position statement, the College declared a climate and ecological emergency that is impacting on the mental health of populations, including the amplification of existing inequalities.^8^ The position statement^8^ describes the intimate connections between health and the natural world; our species is one part of a complex inter-dependent ecosystem. Depression, anxiety and post-traumatic stress disorder are associated with floods.^9^ Suicide is linked to drought and hotter temperatures.^10^ People with severe mental illnesses are three times more likely to die in a heatwave.^11^ Psychiatric medications such as antidepressants and antipsychotics affect heat-regulating functions.^12^ Dehydration and sweating can contribute to lithium toxicity, which places those taking these treatments at increased risk during heatwaves.^12^ Food insecurity affects childhood development.^13^ Air pollution is associated with anxiety and depression,^14^ and neurodevelopmental effects in children,^15^ and accounts for 6% of the modifiable risk for dementia.^16^ Climate change and ecological breakdown leads to poverty, precarity and social unrest, with associated psychological trauma.^17,18^ Extreme weather events are likely to make it increasingly difficult for us to deliver healthcare to communities. The secondary effects of the crisis include climate grief and anxiety, which are particularly noticeable in children and young people.^19^ Their anger, too, is understandable, as they face a future where leaders have delayed action and misinformation continues to permeate mainstream media. A burgeoning field of climate psychology aims to help us understand how we reached this state, face the losses that are already certain and deal with ‘present traumas’.^20^ In the face of such threats, denial of the crisis is a luxury we can no longer afford.

In this special issue, we will learn of the direct effects of extreme weather events and habitat loss, and the resulting social disintegration, on mental health, and the indirect effects of the climate emergency and the sense of insurmountable challenge on our mental well-being. Concepts such as eco-distress, climate grief and solastalgia^21^ (mourning for the degradation of one's home) are explored. We also consider the way forward, and the potential immediate and longer-term health and economic gains in urgent, collective action.

The climate and ecological crisis can trigger a complicated set of psychological reactions, including grief, guilt, fear, anxiety and a fluctuating awareness of our own destructiveness, resulting in varying forms of denial and disavowal.^22^ These responses have been extensively documented by activists and experts in the field of climate psychology. They can, in part, explain why so little action has been taken at the necessary scale and pace, despite repeated promises.^20,23^ Weintrobe describes how the neoliberal mindset of ‘uncare’ and narcissistic entitlement perpetuates ways of living that damage ourselves, our relationships to others and to the planet.^24^ She argues that the more we are able to face the violence beneath our convenient lives, the better we can turn to a more moral path, but courage and kinship is required for facing these realities.^24^

The consequences of ecological destruction fall first and worst on the most marginalised people, including those who are poor, those with severe mental illness, older people and children. Climate and environmental justice is linked to racial justice globally, as the most significant effects of the crisis are already being felt by indigenous peoples and by those in the Global South, who are disadvantaged by colonialism and exploitation. These populations are also least likely to have contributed to the root causes of the climate crisis.

## Healthy solutions

Public health experts have clearly described what must be done to limit the damage. The United Nations Intergovernmental Panel on Climate Change has advised that greenhouse gas emissions need to drop drastically by 2030 to give us any chance of avoiding the most catastrophic effects of global warming.^25^ The *Lancet* Countdown describes the pathway we are on, leading at its current trajectory to a possible 3°C warming, and the second pathway, which aims to keep warming ‘well below’ 2°C above pre-industrial levels.^4^ The reality of those targets is already being felt in all parts of the world. Facing the catastrophe of the climate emergency means putting in place a number of correctives to the way we live today.

At this point, individual action to manage emissions will not produce the rapid decarbonisation and restoration of our habitat that is needed to prevent further loss of life and livelihood. Although many of us will find solace in personal deeds, which are important, an overemphasis on individual behaviour distracts from the collective action that is necessary to face the challenge. We need governments to go beyond goal-setting and begin to deliver on ambitious targets, to take brave and bold steps. But they are unlikely to move far and fast enough without firm instruction, including from healthcare professionals and leaders.

Action to address the causes of the climate crisis improves the well-being of populations, prevents disease, reduces inequalities and is good for the economy.^26^ By reducing use and switching to clean forms of renewable energy, and building active travel infrastructure and sustainable food networks, we create a low-carbon society with enormous health benefits.^27^ Modelling of the potential effect of active travel in England and Wales suggests that increasing walking and cycling could reduce ischaemic heart disease, lung disease, dementia and cancer.^28^ Preserving and increasing biodiverse, unpolluted green and blue spaces carries significant benefits for patients, families, staff and communities.

Embracing our connectedness to the natural world is not only about prevention, but also the potential for healing. Nature-based interventions show a wide range of benefits, including improved well-being and sense of coherence, and reduction of distress and anger in both clinical and non-clinical populations.^29^ The RCPsych statement^8^ highlights the importance of services where staff and patients work together in ‘Choosing Wisley’,^30^ which could reduce waste and replication, and improve sustainability and quality.

## The role of doctors

As engaged and informed doctors have long argued, healthcare professionals need to be aware of, and able to articulate and engage in the politics of the climate and ecological crisis. We are trusted professionals, and as such have an opportunity and, furthermore, a duty to act as leaders in our own organisations and communities. We must be able to communicate the urgency of the problem and the immense benefits of action in preventing unnecessary mortality and morbidity. The National Health Service (NHS) is the single largest source of greenhouse gas emissions in the UK public sector, and 60% of the carbon footprint of the NHS relates to medication and medical equipment.^26^ The Greener NHS report, which sets accelerated targets for a net zero NHS, makes reducing emissions a key responsibility of all NHS staff.^26^ Drivers include professional and patient transport, energy use and consumption, use of natural resources, shifting to preventative practices and reducing unnecessary prescribing.^26^

Increasingly, psychiatrists are raising awareness through direct and coordinated action. Whether through activist groups, such as Doctors for Extinction Rebellion and Psych Declares; through the RCPsych Sustainability Committee; internationally, through the Climate Psychiatry Alliance; or locally, through NHS Trust Green Plans, there are ways for everyone to get involved. Health professionals play an important role in drawing attention to the physical, mental, social and psychological effects of the crisis, and the opportunities offered by its solutions. Our ability to influence government decision-making and public perceptions through awareness is likely to be at least as important as action on reducing our own emissions.

The RCPsych's response to the climate and ecological crisis has been robust and multifaceted. An important step was the February 2020 announcement of divestment from fossil fuels and signing up to the Principles of Responsible Investment supported by the United Nations.^31^ In 2020, a guide and podcast on eco-distress was produced for parents, carers and young people.^19^ A key aspect of these resources is the validation that climate anxiety is not in itself pathological, but an appropriate reaction to the peril we face.

Educating future generations of psychiatrists has been an important focus of the RCPsych's work, and 2020 saw the inclusion of sustainability as a core area of the new curriculum and the launch of a Continuing Professional Development module on sustainable healthcare.^32^

It is, perhaps, younger psychiatrists who best understand the implications of today's climate and ecological crisis. In giving voice to future leaders, the *BJPsych Bulletin* celebrates the winner of the 2020 Praxis Editorial Award, Dr Daniel Romeu, whose entry eloquently argued the importance of action from psychiatrists. The competition received entries from medical students, foundation doctors, trainees, Specialty and Associate Specialist Doctors (SAS) responding to the question ‘Is the climate a mental health crisis?’ They responded with an emphatic 'yes'. We were impressed with their creativity, passion and hope for improving individual and planetary health and addressing inequalities. Congratulations also to the highly commended authors Dr Karyn Ayre, Mr James Street, Dr Fergus Brown and Dr Kris Roberts. We are grateful to our panel of judges: Anouchka Grose, Professor Alex Ford and Dr Katherine Kennet.

## Conclusions

Psychiatrists are in a position to use their collective voice, medical expertise and understanding of both individual and systemic factors, to advocate for redressing inequalities and lead organisational change. The climate crisis amplifies existing health disparities, and disproportionately affects those already vulnerable owing to poverty or underlying conditions.

This special issue of the *BJPsych Bulletin* contributes to the compelling argument that the climate crisis is a mental health crisis, and that working to redress this should become core business for psychiatrists. Not only must this climate crisis issue induce alarm, but it should also generate hope, resolve and action.

Sally Weintrobe reflected in a recent paper, ‘People, young and old, are at the point of beginning to find the collective courage to face the shock required to emerge from our retreat from reality’.^24^ We trust these papers motivate and inspire you not only to face the reality of this emergency, but to engage creatively with action that improves the health of the populations you serve, and the environment in which you live and work.

## About the authors

**Dr Cate Bailey** is an ST7 General Adult and Old Age Psychiatrist with the East London NHS Foundation Trust, UK; Honorary Clinical Lecturer with Barts and the London School of Medicine, UK; Trainees’ Section Editor of the *BJPsych Bulletin*, on behalf of the Royal College of Psychiatrists, UK; and co-Chair of the Older Adults Section of the Association for Psychoanalytic Psychotherapy in the NHS, UK. **Dr Norman A. Poole** is Editor-in-Chief of the *BJPsych Bulletin*, on behalf of the Royal College of Psychiatrists, UK; and a Consultant Neuropsychiatrist at St George's Hospital, South West London and St George's Mental Health NHS Trust, UK. **Dr Adrian James** is President of the Royal College of Psychiatrists, UK.

## References

[1] Wray B. *Using Grief, Rage and Dread to Collectively Wake Up from the Trance of Denial: A Conversation with Activist, Psychologist and Author Margaret Klein Salamon.* GenDread. 2021 (https://gendread.substack.com/p/using-grief-rage-and-dread-to-collectively).

[2] NHS England. *NHS Becomes the World's First National Health System to Commit to Become ‘Carbon Net Zero’, Backed by Clear Deliverables and Milestones.* NHS England, 2020 (https://www.england.nhs.uk/2020/10/nhs-becomes-the-worlds-national-health-system-to-commit-to-become-carbon-net-zero-backed-by-clear-deliverables-and-milestones/).

[3] World Health Organization (WHO). *Quantitative Risk Assessment of the Effects of Climate Change on Selected Causes of Death, 2030s and 2050s.* WHO, 2014 (http://www.who.int/globalchange/publications/quantitative-risk-assessment/en/).

[4] Watts N, Amann M, Arnell N, Ayeb-Karlsson S, Beagley J, Belesova K, The 2020 report of The Lancet Countdown on health and climate change: responding to converging crises. Lancet 2021; 397: 129–70. 33278353 10.1016/S0140-6736(20)32290-X PMC7616803

[5] Godlee F. Climate change. BMJ 2014; 349: g5945. 10.1136/bmj.g5945 25274623

[6] Haines A, Scheelbeek P, Abbasi K. Challenges for health in the Anthropocene epoch. BMJ 2019; 364: 2019–20. 10.1136/bmj.l460 PMC7616371 30718363

[7] UK Health Alliance on Climate Change. *UK Health Alliance on Climate Change Response to the Greener NHS Consultation*. UK Health Alliance on Climate Change, 2020 (http://www.ukhealthalliance.org/wp-content/uploads/2020/03/GreenerNHS-UKHACC-Response-online.pdf).

[8] Royal College of Psychiatrists. PS03/21: Our Planet’s Climate and Ecological Emergency. Royal College of Psychiatrists, 2021 (https://www.rcpsych.ac.uk/docs/default-source/improving-care/better-mh-policy/position-statements/position-statement-ps03-21-climate-and-ecological-emergencies-2021.pdf).

[9] Waite TD, Chaintarli K, Beck CR, Bone A, Amlôt R, Kovats S, The English national cohort study of flooding and health: cross-sectional analysis of mental health outcomes at year one. BMC Public Health 2017; 17: 129. 28129752 10.1186/s12889-016-4000-2 PMC5273816

[10] Hanigan IC, Butler CD, Kokic PN, Hutchinson MF. Suicide and drought in New South Wales, Australia, 1970–2007. Proc Natl Acad Sci USA 2012; 109(35): 13950–5. 22891347 10.1073/pnas.1112965109 PMC3435226

[11] Bouchama A, Dehbi M, Mohamed G, Matthies F, Shoukri M, Menne B. Prognostic factors in heat wave – related deaths. Arch Intern Med 2012; 167(20): 2170–6. 10.1001/archinte.167.20.ira70009 17698676

[12] Martin-Latry K, Goumy MP, Latry P, Gabinski C, Bégaud B, Faure I, Psychotropic drugs use and risk of heat-related hospitalisation. Eur Psychiatry 2007; 22(6): 335–8. 17513091 10.1016/j.eurpsy.2007.03.007

[13] Carter KN, Kruse K, Blakely T, Collings S. The association of food security with psychological distress in New Zealand and any gender differences. Soc Sci Med 2011; 72(9): 1463–71. 21481507 10.1016/j.socscimed.2011.03.009

[14] Braithwaite I, Zhang S, Kirkbride JB, Osborn DPJ, Hayes JF. Air pollution (particulate matter) exposure and associations with depression, anxiety, bipolar, psychosis and suicide risk: a systematic review and meta-analysis. Environ Health Perspect 2019; 127(12): 126002. 31850801 10.1289/EHP4595 PMC6957283

[15] Payne-Sturges DC, Marty MA, Perera F, Miller MD, Swanson M, Ellickson K, Healthy air, healthy brains: advancing air pollution policy to protect children's health. Am J Public Health 2019; 109(4): 550–4. 30789769 10.2105/AJPH.2018.304902 PMC6417586

[16] Livingston G, Huntley J, Sommerlad A, Ames D, Ballard C, Banerjee S, Dementia prevention, intervention, and care: 2020 report of the Lancet Commission. Lancet 2020; 396(10248): 413–46. 32738937 10.1016/S0140-6736(20)30367-6 PMC7392084

[17] Marotzke J, Semmann D, Milinski M. The economic interaction between climate change mitigation, climate migration and poverty. Nat Clim Chang 2020; 10(6): 518–25.

[18] Torres JM, Casey JA. The centrality of social ties to climate migration and mental health. BMC Public Health 2017; 17: 600. 28679398 10.1186/s12889-017-4508-0 PMC5498922

[19] Royal College of Psychiatrists. The Climate Crisis Is Taking a Toll on the Mental Health of Children and Young People. Royal College of Psychiatrists, 2020 (https://www.rcpsych.ac.uk/news-and-features/latest-news/detail/2020/11/20/the-climate-crisis-is-taking-a-toll-on-the-mental-health-of-children-and-young-people).

[20] Harvey A, Manley J, Hickman C. Ecology, psychoanalysis and global warming: present and future traumas. J Soc Work Pract 2020; 34(4): 337–48.

[21] Albrecht G, Sartore GM, Connor L, Higginbotham N, Freeman S, Kelly B, Solastalgia: the distress caused by environmental change. Australas Psychiatry 2007; 15(suppl 1): 95–8. 10.1080/10398560701701288 18027145

[22] Weintrobe S. Introduction. In Engaging with Climate Change Psychoanalytic and Interdisciplinary Perspectives (1st edn) (ed. S Weintrobe): 1–15. Routledge, 2012.

[23] Brenner I. Climate change and the human factor: why does not everyone realize what is happening? Int J Appl Psychoanal Stud 2019; 16(2): 137–43.

[24] Weintrobe S. Moral injury, the culture of uncare and the climate bubble. J Soc Work Pract 2020; 34(4): 351–62.

[25] Masson-Delmotte V, Zhai P, Pörtner H-O, Roberts D, Skea PR, Shukla A, *Summary for Policymakers. Global Warming of 1.5°C. An IPCC Special Report on the Impacts of Global Warming of 1.5°C Above Pre-industrial Levels and Related Global Greenhouse Gas Emission Pathways, in the Context of Strengthening the Global Response to the Threat of Climate Change, Sustainable Development, and Efforts to Eradicate Poverty.* Intergovernmental Panel on Climate Change, 2018 (https://www.ipcc.ch/site/assets/uploads/sites/2/2019/05/SR15_SPM_version_report_LR.pdf).

[26] The NHS Net Zero Expert Panel. Delivering a “Net Zero” National Health Service. NHS England and NHS Improvement, 2020 (https://www.england.nhs.uk/greenernhs/wp-content/uploads/sites/51/2020/10/delivering-a-net-zero-national-health-service.pdf).

[27] Milner J, Hamilton I, Woodcock J, Williams M, Davies M, Wilkinson P, Health benefits of policies to reduce carbon emissions. BMJ 2020; 368: 6–11. 10.1136/bmj.l6758 PMC7190375 32229476

[28] Woodcock J, Givoni M, Morgan AS. Health impact modelling of active travel visions for England and Wales using an integrated transport and health impact modelling tool (ITHIM). PLOS One 2013; 8(1): e51462. 23326315 10.1371/journal.pone.0051462 PMC3541403

[29] Masterton W, Carver H, Parkes T, Park K. Greenspace interventions for mental health in clinical and non-clinical populations: what works, for whom, and in what circumstances? Heal Place 2020; 64: 102338. 10.1016/j.healthplace.2020.102338 32838901

[30] Maughan D, James A. Diagnosis and treatment: are psychiatrists choosing wisely? BJPsych Adv 2017; 23(1): 9–15.

[31] Royal College of Psychiatrists. College Leads the Way in Responsible Investment. Royal College of Psychiatrists, 2020 (https://www.rcpsych.ac.uk/members/your-monthly-enewsletter/rcpsych-enewsletter-february-2020/college-goes-greener).

[32] Richardson J, Grant-Peterkin H, Maughan D. *Sustainability: A Key Concept in Leadership and Service Development*. CPD Online, 2020 (https://elearning.rcpsych.ac.uk/learningmodules/sustainabilityakeyconcept.aspx).

